# Serum uric acid and the incidence of CKD and hypertension

**DOI:** 10.1007/s10157-015-1120-4

**Published:** 2015-05-13

**Authors:** Satoru Kuriyama, Yukio Maruyama, Shinichiro Nishio, Yasuhito Takahashi, Satoshi Kidoguchi, Chisa Kobayashi, Daisuke Takahashi, Naoki Sugano, Tatsuo Hosoya, Takashi Yokoo

**Affiliations:** Department of Pathophysiology and Therapy in Chronic Kidney Disease, Jikei University School of Medicine, Tokyo, Japan; Division of Nephrology and Hypertension, Department of Internal Medicine, Jikei University School of Medicine, 3-25-8, Nishi-shinbashi, Minato-ku, Tokyo, 105-8461 Japan

**Keywords:** Uric acid, CKD, Hypertension, Estimated glomerular filtration rate

## Abstract

**Background:**

Uric acid (UA) levels correlate positively with the prevalence of chronic kidney disease (CKD) and/or hypertension. We tested the hypothesis that UA may also have a link to a new incidence of CKD and hypertension.

**Methods:**

Study design is a cohort study and the predictor is UA levels. Of the 15,470 screened cases, 8223 participants without CKD were eligible for the analysis of the incidence of CKD. Among these CKD candidates, 7569 participants were eligible for the analysis of the new development of hypertension. The observation period was 4 years.

**Results:**

Relationship of UA with new cases of CKD. Higher UA levels had a closer association with the new development of CKD; 1.1 % (UA < 5 mg/dL), 1.5 % (5.0–5.9 mg/dL), 1.7 % (6.0–6.9 mg/dL), and 3.4 % (≧7 mg/dL), respectively (*p* < 0.001 by the Chi-square test). Cox proportional hazard analysis showed that the estimates of the CKD development were eGFR [Hazard Ratio (HR) 0.816, 95 % confidence intervals (CI) 0.791–0.840] and male gender (HR 0.562, 95 % CI 0.322–0.982). UA levels and new development of hypertension. Higher UA levels had a closer association with the new development of hypertension; 5.0 % (UA < 5 mg/dL), 8.9 % (5.0–5.9 mg/dL), 10.6 % (6.0–6.9 mg/dL), and 11.8 % (≧7 mg/dL), respectively (*p* < 0.001 by the Chi-square test). Cox proportional hazard analysis showed that the estimates of the hypertension development were BMI (HR 1.190, 95 % CI 1.155–1.226), age (HR 1.021, 95 % CI 1.010–1.032), HDL-cholesterol (HR 1.013, 95 % CI 1.007–1.019), male gender (HR 1.791, 95 % CI 1.338–2.395), UA level (HR 1.112, 95 % CI 1.024–1.207), and eGFR (HR 1008, 95 % CI 1.002–1.013). Furthermore, the logistic analysis showed that the odds ratio (OR) to estimate hypertension in the high UA group (UA ≧ 7 mg/dL; OR 1.33, 95 % CI 1.01–1.80) was greater than that in the low UA group (UA < 5 mg/dL). Kaplan–Meier analysis also confirmed the finding that the higher the UA levels the greater the hypertension development (*p* < 0.001 by the Log-rank test and Cox proportional hazard analysis).

**Conclusion:**

High UA levels are associated with the new development of hypertension, but not with the incidence of CKD.

## Introduction

Previous studies showed that circulating high uric acid (UA) levels were associated with increased prevalence of hypertension and a high risk status of cardiovascular complications which frequently leads to poor patient prognosis [1–6]. Increased UA levels may also play a pivotal role in the progression of chronic kidney disease (CKD) such as chronic glomerulonephritis [7], diabetic nephropathy [5, 8], end stage renal failure [7, 8], and gouty kidney [9]. These data are suggestive of UA as a precipitating factor in the progression of CKD and/or hypertension. The potential mechanisms to account for these associations may be diverse; i.e., endothelial dysfunction, the activation of the intrarenal renin-angiotensin system, a vascular smooth muscle cell proliferation, the increased synthesis of interleukin-6, insulin resistance, and impaired endothelial nitric oxide productions [10]. Regardless of these affirmative reports, the relationship between UA levels and CKD or hypertension is not always consistent. In fact, some other studies performed in other countries did not support such an association between kidney dysfunction and hyperuricemia [11–14]. At this point, therefore, one can conclude the role of hyperuricemia in the CKD progression and hypertension in humans is still a matter of controversy. Regarding the relationship of early onset with UA level, evidences are much more scant. Thus, further studies to investigate whether UA can trigger the early onset of CKD and/or hypertension are needed to address this question.

In the present study, with the above-mentioned background, we carried out a longitudinal retrospective survey to look into the associations of UA level with the development of hypertension and the new onset of CKD in a screened population at large. We applied strict exclusion criteria in order to choose eligible participants who did not have a concurrent or a past history of CKD and/or hypertension.

## Subjects and methods

### Study population and design

The study design is a retrospective population-based cohort of Japanese office workers aged 25–60 years, living in the vicinity of Tokyo. The original number of the participants was 15,470 who had an annual medical check-up every year from 2008 to 2012. They were seen at least once a year by well-trained physicians or public nurses.

The basic criteria excludes individuals receiving medications for diabetes, hyperuricemia, hypertension, and dyslipidemia throughout the follow-up period of 4 years; those having insufficient data; those with past history of incident major cardiovascular events (MACE) such as cerebral apoplexy or myocardial infarction; those with any disease requiring hospitalization; those with current cancer or other life-threatening diseases; those with current pregnancy, and those with estimated glomerular filtration rate (eGFR) less than 60 mL/min/1.73 m^2^. The present and past history of proteinuria was also another rule-out factor. This exclusion process finally leaves 8223 for the analysis of the new onset of CKD. Finally, the additional application of exclusion criterion of a blood pressure (BP) rise to systolic of 140 mmHg or greater, and diastolic of 90 mmHg or greater at the beginning leaves 7569 individuals for the analyses of the new development of hypertension.

### Primary predictor and outcome

The primary predictor was the level of UA. UA levels were divided into 4 groups (Group 1: UA < 5.0 mg/dL, Group 2: UA 5.0–5.9 mg/dL, Group 3: UA 6.0–6.9 mg/dL, Group 4: UA ≧ 7.0 mg/dL) and were evaluated in conjunction with newly developed CKD and hypertension. The new incidence of CKD was defined as a decline in eGFR to less than 60 mL/min/1.73 m^2^ calculated each year. Similarly, the new onset of hypertension was defined as a rise of systolic blood pressure (SBP) to 140 mmHg or greater, and/or diastolic blood pressure (DBP) of 90 mmHg or greater evaluated at each year. Renal function expressed as eGFR for Japanese was calculated based on the equation; eGFR = 194 × Cr^−1.094^ × Age^−0.287^ (if women × 0.739), reported elsewhere [15].

### Other variables

Body mass index (BMI) was calculated based on the equation; BMI = Body weight (BW) × 1/(Body Height) squared. Laboratory tests were carried out after an 8-to 12-h fast. Measurements were made on serum creatinine (Cr) concentration, serum uric acid (UA) concentration, blood urea nitrogen (BUN), electrolytes, and lipid profiles including total cholesterol (TC), triglycerides (TG), high-density lipoprotein cholesterol (HDLC), low-density lipoprotein cholesterol (LDLC), HbA1c, and plasma glucose (PG). Laboratory tests were performed using the Toshiba auto-analyzer (TBA-80 FR neo, Tokyo, Japan). Urinalysis was performed for qualitative measurement of urinary protein. Quantitative measurement of daily urinary protein excretion was not available. The value of HbA1c was measured as a unit of JDS (Japan Diabetes Society). Following the worldwide recommendations, all of the HbA1c values were expressed as a unit of NGSP (National glycohemoglobin standardization program) based on the equation; NGSP (%) = JSD × 1.02 + 0.25 (%).

Blood pressure (BP) was measured in a sitting position during a morning visit (fasting, 9–11 AM), after 5 min of rest in the supine position by an automatic self-measuring device equipped with a 47 × 13 cm cuff and 24 × 13 cm bladder to avoid so-called “white coat hypertension” and/or “cuff hypertension”.

### Ethical considerations

The present study was conducted in accordance with “Recommendations on the Establishment of Animal Experimental Guidelines” approved at the 80th General Assembly of the Japanese Science Council in 1980, and the principles set out in the Declaration of Helsinki 1964 as modified by subsequent revisions.

The study protocol design was a retrospective screened cohort. This epidemiological survey was submitted to the Institutional Review Board (IRB)/Ethics Committee of the Jikei University School of Medicine. After the deliberation the protocol was approved by the ethical committee of the University with the clinical trial number 25-203 (7338).

### Statistical analysis

Uric acid was analyzed by dividing the distribution into 4 groups. Cross-sectional associations of baseline demographics and risk factors with UA groups were performed using Chi-square analysis for discrete variables, analysis of variance (ANOVA) for continuous variables. *p* values were calculated for linear trend across the 4 UA groups. Cox proportional hazard analysis was used to estimate adjusted hazard ratio (HR) and associated 95 % confidence intervals (CI) for either CKD onset or hypertension development. In the longitudinal analysis, we examined the association of new incident CKD and new onset of hypertension according to baseline UA levels. For these dichotomous outcomes, logistic regression analysis was used to estimate adjusted odds ratio (OR) and associated 95 % CI in relation to 4 UA groups. UA was modeled as both a linear variable per 1 mg/dL increase.

Variables significant in univariate analyses were considered to be potential confounders of UA in multivariate models. The final model was adjusted for age, gender, BMI, baseline serum Cr concentration, SBP and DBP, HbA1c and lipid levels. The final variables were chosen on the basis of biological plausibility at the doctor’s discretion. After obtaining the statistical significance as the estimate in the multivariate analysis, univariate analysis to estimate time-to-disease-free curves in relation to the UA levels was performed using Kaplan–Meier method and compared with stratified Log-rank tests. In addition, Cox proportional hazard analysis was employed to evaluate the statistical difference among the 4 UA groups.

Statistical analyses were carried out with Stat Flex version 6.0 (Artec Ltd. Co., Osaka, Japan). Data are presented as the mean ± standard deviation (SD), unless otherwise indicated. *p* < 0.05 is considered statistically significant. Confidence intervals (CI) are expressed as 95 % CI.

## Results

### CKD analysis

#### Baseline Characteristics

The study cohort had an average age of 39 ± 10 years, an eGFR of 91.2 ± 17.6 mL/min/1.73 m^2^, a UA level of 5.7 ± 1.4 mg/dL, after the exclusion at the start, and the follow-up term was 4 years. Participants were all native Japanese. Greater UA levels were significantly associated with older age, males, greater prevalence of hypertension, BMI, waist circumference, SBP, DBP, serum Cr concentration, TC, TG, HDLC, LDLC, PG, HbA1c, and lesser eGFR (Table 1).

**Table 1:** Demographics of subjects for CKD onset

	Uric acid groups (mg/dL)					
	Overall	<5.0	5.0–5.9	6.0–6.9	≧7.0	*p*
*N*	8223	2631	2418	2118	1056	
Age (years)	39 ± 10	37 ± 9	39 ± 10	40 ± 10	41 ± 9	<0.001
Men (%)	77.7	42.2	89.6	97.9	98.8	<0.001
Hypertension (%)	7.7	3.8	7.1	10.6	15.1	<0.001
BMI (Kg/m^2^)	22 ± 3	21 ± 3	22 ± 3	23 ± 3	24 ± 3	<0.001
Waist circumference (cm)	78.6 ± 8.8	74.1 ± 8.2	78.6 ± 7.9	81.1 ± 7.9	84.2 ± 8.6	<0.001
SBP (mmHg)	118 ± 13	113 ± 12	118 ± 12	120 ± 13	123 ± 14	<0.001
DBP (mmHg)	74 ± 9	70 ± 9	74 ± 9	75 ± 9	77 ± 9	<0.001
Cr (mg/dL)	0.75 ± 0.14	0.65 ± 0.13	0.77 ± 0.11	0.80 ± 0.11	0.83 ± 0.11	<0.001
eGFR (mL/min/1.73 m^2^)	91.2 ± 17.6	96.5 ± 19.5	91.1 ± 16.6	88.1 ± 15.4	84.3 ± 15.0	<0.001
TC (mg/dL)	192 ± 29	187 ± 29	192 ± 29	194 ± 28	201 ± 28	<0.001
TG (mg/dL)	94 ± 59	72 ± 42	91 ± 51	106 ± 65	126 ± 76	<0.001
HDLC (mg/dL)	64 ± 15	69 ± 15	64 ± 15	61 ± 14	59 ± 16	<0.001
LDLC (mg/dL)	113 ± 26	106 ± 25	113 ± 26	117 ± 26	122 ± 27	<0.001
PG (mg/dL)	93 ± 10	91 ± 9	93 ± 10	94 ± 10	96 ± 11	<0.001
HbA1c (NGSP) (%)	5.23 ± 0.34	5.21 ± 0.33	5.23 ± 0.33	5.25 ± 0.34	5.29 ± 0.37	<0.001

*UA* serum uric acid concentration, *BMI* body mass index, *SBP* systolic blood pressure, *DBP* diastolic blood pressure, *Cr* serum creatinine concentration, *eGFR* estimated glomerular filtration rate, *TC* total cholesterol, *TG* triglycerides, *HDLC* high-density lipoprotein cholesterol, *LDLC* low-density lipoprotein cholesterol, *PG* plasma glucose concentration, *HbA1c* glycated hemoglobin

#### Incidence of CKD

**Fig. 1:**
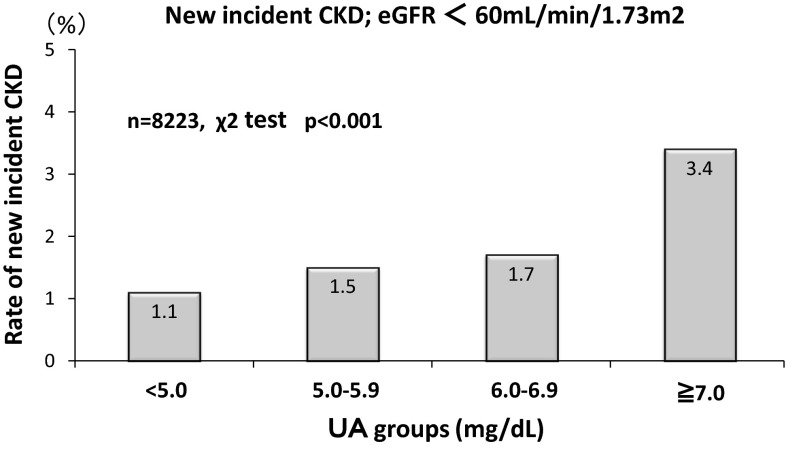
New incidence of CKD at year 4. Newly onset of CKD was depicted at year 4 according to UA groups. The incidence rate increases significantly as UA levels increases (*p* < 0.001 by the Chi-square analysis)

Figure 1 showed the incident rates of newly developed CKD in a period of 4 years. Higher UA levels were significantly associated with greater incidence of CKD, 1.1 % (UA < 5.0 mg/dL), 1.5 % (5.0–5.9 mg/dL), 1.7 % (6.0–6.9 mg/dL), and 3.4 % (≧7.0 mg/dL), respectively (by the Chi-square test, *p* < 0.001). The greater UA levels are associated with the higher incidence of CKD. This increasing trend is especially prominent in the individuals group with the highest UA level (UA ≧ 7 mg/dL). A total of 139 individuals had a decrease in eGFR less than 60 mL/min/1.73 m^2^ at year 4 (139/8223 individuals = 1.7 %). Among them, the rate of proteinuria at year 4 was 1.4 % (2/139 individuals).

#### Association of CKD onset with independent variables

Table 2 showed various parameters that were associated the incidence of CKD, defined as a decline in eGFR less than 60 mL/min/1.73 m^2^ at year 4. The Cox proportional hazard analysis shows that the estimates were eGFR [Hazard Ratio (HR) 0.816, 95 % confidence intervals (CI) 0.791–0.840] and male gender (HR 0.562, 95 % CI 0.322–0.982), suggesting that UA is not chosen as an estimate to predict the incident CKD in this multivariate analysis.

**Table 2:** Cox proportional hazard analysis to estimate the CKD onset

Variable	*β*	SE (*β*)	HR	95 % CI	*p*
eGFR	−0.204	0.015	0.816	0.791–0.840	<0.001
Male	−0.576	0.285	0.562	0.322–0.982	0.043
SBP	0.021	0.011	1.021	0.999–1.043	0.058
DBP	−0.023	0.016	0.977	0.947–1.008	0.151
Age	0.019	0.014	1.019	0.991–1.047	0.187
TC	−0.004	0.004	0.996	0.989–1.002	0.202
TG	0.002	0.002	1.002	0.999–1.004	0.307
UA	0.080	0.090	1.083	0.909–1.290	0.375
HbA1c	0.141	0.289	1.152	0.654–2.027	0.624
BMI	0.014	0.035	1.014	0.947–1.087	0.686

Cox proportional hazard analysis was employed to identify factors to explain the onset of CKD

#### Longitudinal outcome of CKD onset

In 139 individuals with CKD at year 4, mean eGFR at baseline and closeout were 67.7 ± 7.2 and 57.2 ± 2.5 mL/min/1.73 m^2^, respectively. In contrast, mean eGFR of individuals without CKD incidence at baseline and closeout were 91.6 ± 17.5 and 84.4 ± 13.7 mL/min/1.73 m^2^ (*n* = 8084), respectively.

### Hypertension analysis

#### Baseline characteristics

Participants with hypertension at the start were excluded from the population for CKD analysis, eliminating 654 participants from 8223, leaving 7569 participants for the hypertension analysis. This study group had an average age 39 ± 10 years, UA level 5.6 ± 1.3 mg/dL at the start. Greater UA levels were associated with older age, males, greater BMI, waist circumference, SBP, DBP, serum Cr concentration, TC, TG, HDLC, LDLC, PG, HbA1c, and lesser eGFR (Table 3).

**Table 3:** Demographics of subjects for hypertension development

	Uric acid groups (mg/dL)					
	Overall	<5.0	5.0–5.9	6.0–6.9	≧7.0	*p*
*N*	7569	2531	2247	1894	897	
Age (years)	39 ± 10	37 ± 9	39 ± 10	40 ± 9	41 ± 9	<0.001
Men (%)	76.3	40.9	89.2	97.8	98.8	<0.001
BMI (Kg/m^2^)	22 ± 3	21 ± 3	22 ± 3	23 ± 3	24 ± 3	<0.001
Waist circumference (cm)	78.1 ± 8.6	73.9 ± 8.1	78.3 ± 7.8	80.8 ± 7.8	83.7 ± 8.1	<0.001
SBP (mmHg)	115 ± 11	112 ± 11	116 ± 11	117 ± 10	119 ± 10	<0.001
DBP (mmHg)	72 ± 8	70 ± 8	73 ± 8	74 ± 8	75 ± 7	<0.001
Cr (mg/dL)	0.75 ± 0.14	0.65 ± 0.13	0.77 ± 0.11	0.80 ± 0.11	0.83 ± 0.11	<0.001
eGFR (mL/min/1.73 m^2^)	91.4 ± 17.7	96.5 ± 19.6	91.2 ± 16.7	88.1 ± 15.3	84.1 ± 15.0	<0.001
TC (mg/dL)	191 ± 29	187 ± 28	191 ± 29	194 ± 28	201 ± 29	<0.001
TG (mg/dL)	91 ± 57	71 ± 41	91 ± 51	104 ± 62	125 ± 76	<0.001
HDLC (mg/dL)	64 ± 15	69 ± 15	63 ± 15	61 ± 14	59 ± 15	<0.001
LDLC (mg/dL)	112 ± 26	106 ± 25	113 ± 26	116 ± 26	122 ± 27	<0.001
PG (mg/dL)	93 ± 9	91 ± 9	93 ± 10	94 ± 9	96 ± 10	<0.001
HbA1c (NGSP) (%)	5.23 ± 0.34	5.20 ± 0.33	5.23 ± 0.32	5.25 ± 0.34	5.29 ± 0.37	<0.001

*UA* serum uric acid concentration, *BMI* body mass index, *SBP* systolic blood pressure, *DBP* diastolic blood pressure, *Cr* serum creatinine concentration, *eGFR* estimated glomerular filtration rate, *TC* total cholesterol, *TG* triglycerides, *HDLC* high-density lipoprotein cholesterol, *LDLC* low-density lipoprotein cholesterol, *PG* plasma glucose concentration, *HbA1c* glycated hemoglobin

#### Incidence of newly–developed hypertension

**Fig. 2:**
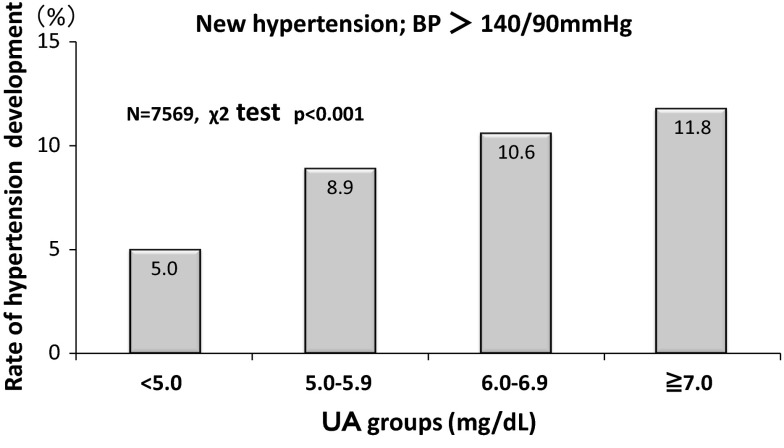
New development of hypertension at year 4. New onset of hypertension was depicted at year 4 according to UA groups. The incidence rate increases significantly as UA levels increases (*p* < 0.001 by the Chi-square analysis)

Figure 2 showed the incident rates of newly developed hypertension within a period of 4 years. Higher UA levels had a close association with the new hypertension; 5.0 % (UA < 5.0 mg/dL), 8.9 % (5.0–5.9 mg/dL), 10.6 % (6.0–6.9 mg/dL), and 11.8 % (≧7.0 mg/dL), respectively (*p* < 0.001 by the Chi-square test). The greater incidence remained linearly associated with increasing UA level. A total of 633 individuals developed hypertension (633/7569 individuals = 8.4 %). Among them, the rate of proteinuria at year 4 was 1.6 % (10/633 individuals). In addition, among 633 individuals who became hypertensives, there were 13 individuals who developed both CKD and hypertension at year 4 (data not shown).

#### Association of hypertension onset with independent variables

Table 4 showed various parameters that were associated with the onset of hypertension defined as a rise of SBP to 140 mmHg or greater, DBP of 90 mmHg or greater at year 4. Cox proportional hazard analysis shows that such variables were BMI (HR 1.190, 95 % CI 1.155–1.226), age (HR 1.021, 95 % CI 1.010–1.032), HDL-cholesterol (HR 1.013, 95 % CI 1.007–1.019), male gender (HR 1.791, 95 % CI 1.338–2.395), UA level (HR 1.112, 95 % CI 1.024–1.207), and eGFR (HR 1.008, 95 % CI 1.002–1.013).

**Table 4:** Cox proportional hazard analysis to estimate the hypertension development

Variable	*β*	SE (*β*)	HR	95 % CI	*p*
BMI	0.174	0.015	1.190	1.155–1.226	<0.001
Age	0.021	0.006	1.021	1.010–1.032	<0.001
HDLC	0.014	0.003	1.013	1.007–1.019	<0.001
Male	0.583	0.149	1.791	1.338–2.395	<0.001
UA	0.106	0.042	1.112	1.024–1.207	0.012
eGFR	0.008	0.003	1.008	1.002–1.013	0.012
TG	0.001	0.001	1.000	0.999–1.002	0.319
HbA1c	−0.053	0.147	0.948	0.711–1.265	0.718

Cox proportional hazard analysis was employed to identify factors to explain the onset of CKD. BMI, Age, Sex, HDLC, and UA were chosen as such independent variables

#### Longitudinal relationship between UA groups and hypertension

In 633 individuals with hypertension at year 4, the BP at baseline and closeout were 124.2 ± 8.4 mmHg in SBP, 78.3 ± 6.7 mmHg in DBP and 140.7 ± 10.0 mmHg in SBP, 91.6 ± 7.1 mmHg in DBP, respectively. Mean eGFR at baseline and closeout were 89.5 ± 16.9 and 83.5 ± 13.7 mL/min/1.73 m^2^, respectively. In contrast, mean eGFR of individuals without developing hypertension within 4 years (*n* = 6936) at baseline and closeout were 91.5 ± 17.8 and 84.1 ± 14.1 mL/min/1.73 m^2^, respectively. Greater UA levels were associated with greater OR of hypertension development. After adjustment for age, sex, BMI, waist circumference, SBP, DBP, TC, TG, HDLC, LDLC, PG, and HbA1c, multivariate adjusted OR of the logistic regression analysis for new onset of hypertension was 1.00 (reference), 1.30 (95 % CI 1.00–1.69), 1.38 (95 % CI 1.05–1.81), and 1.33 (95 % CI 1.01–1.80) for Group 1 through Group 4, respectively (Table 5). All three groups of UA levels (Group 2, 3, and 4) remained significantly associated with newly developed hypertension.

**Table 5:** Logistic regression analysis to estimate the hypertension development

Uric acid groups (mg/dL)				
	<5.0	5.0–5.9	6.0–6.9	≧7.0
Development of hypertension (*n* = 633; 8.4 %)	127 (20.1 %)	199 (31.4 %)	201 (31.8 %)	106 (16.7 %)
Unadjusted OR (95 % CI)	1.00 (reference)	1.84 (1.46–2.32)	2.25 (1.78–2.83)	2.54 (1.94–3.32)
Adjusted OR (95 %CI)	1.00 (reference)	1.30 (1.00–1.69)	1.38 (1.05–1.81)	1.33 (1.01–1.80)

The development of hypertension was defined as a BP rise more than 140/90 mmHg in systolic and/or diastolic at year 4. The OR was adjusted for age, sex, BMI, waist circumference, SBP, DBP, TC, TG, HDLC, LDLC, PG, and HbA1c

#### Association between hypertension development and UA

**Fig. 3:**
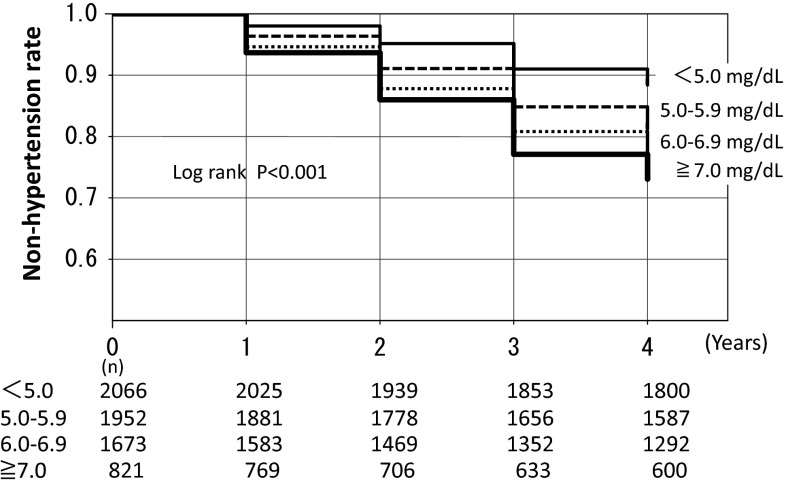
Hypertension-free rate according to UA groups. The occurrence of hypertension in 4 years was presented according to UA groups. Each *line* has proven statistically different by the Log-rank test (*p* < 0.001)

Figure 3 showed Kaplan–Meier analysis to indicate hypertension-free rates according to UA groups. The survival from hypertension at year 4 was calculated as a function of each UA group. There was a statistical difference in the hypertension-free rate among the 4 groups with different UA levels (the Log-rank test, *p* < 0.001). Furthermore, multivariate Cox proportional hazard analysis shows that the UA levels to be associated with hypertension development were Group 2 (UA: 5.0–5.9 mg/dL, HR 1.382, 95 % CI 1.152–1.658), Group 3 (UA: 6.0–6.9 mg/dL, HR 1.673, 95 % CI 1.389–1.658), and Group 4 (UA ≧ 7.0 mg/dL, HR 2.006, 95 % CI 1.634–2.463) as a reference of Group 1 (UA < 5.0 mg/dL).

## Discussion

The present study demonstrates that UA levels were associated with the development of hypertension, but not with the incidence of CKD. The strengths of this study are the large number of individuals who did not have CKD or hypertension at the entry, the complete nature of the data set, and the ability to link demographic and clinical factors with CKD and rise in BP. Other advantages include no new medications for hyperuricemia, diabetes, hypertension, and dyslipidemia throughout the observation period and the ability to adjust for multiple factors that may affect initiation of CKD and hypertension. We also controlled for the currently treated diabetes, hypertension, hyperuricemia, dyslipidemia, and MACE etc., all known as well-established predictors of kidney dysfunction.

### Association of UA levels with CKD incidence

Increased UA is associated with increased risk for future hypertension, diabetic nephropathy, and CKD, and that hyperuricemia, per se, could be an independent risk factor for poor cardiovascular prognosis [1–8, 16, 17]. Advanced CKD frequently induces fatal cardiovascular complications [12]. UA level is an independent risk factor for the progression of kidney disease in a variety of kidney diseases [5–9, 16, 17]. In addition, a prospective controlled trial examined the clinical effect of decreasing UA level with allopurinol in patients with CKD and hyperuricemia [18]. The study disclosed that subjects in the UA-lowering group showed less kidney dysfunction, suggesting a role of UA in the pathogenesis of CKD.

The cause of CKD is multiple. Thus, it is of interest to look into factors that have impact on both the initiation and the progression of CKD in conjunction with UA levels. So far, the role of UA on the incidence of CKD is controversial [12–14, 18–21]. Weiner et al. [20] recruited 13,338 participants with intact kidney function in two community-based cohorts and concluded that elevated UA level is a modest, independent risk factor for incident CKD. Furthermore, a meta-analysis consisting of 15 cohorts with a recruitment of 99,205 individuals and 3492 incident CKD cases showed that the relative risk of CKD was 1.22 (95 % CI 1.16–1.28) per 1 mg/dL serum UA level increment, suggesting a positive link between the two [22].

The present study demonstrates that after the adjustment of confounding factors by the multivariate analysis, UA is no longer an estimate to predict the incident CKD (Table 2), despite the positive link between the incidence rates and the levels of UA (Fig. 1). It is apparent that UA levels are greatly influenced by the confounding effect of eGFR. The involvement of UA on the CKD incidence, in this context, could be difficult to detect in an epidemiological study. We can conclude, at this point, that high UA does not act as a trigger for new incidence of CKD.

### Association of UA levels with hypertension development

Animal experiments demonstrate that increased serum UA causes hypertension that is reversible at the early stage but becomes irreversible at the later stage [9]. In human, substantial number of literature reviews shows a dose-dependent, linear consistent relationship of UA level with BP [23–29]. A total of 18 prospective cohort studies representing data from 55,607 participants clearly demonstrate that hyperuricemia was associated with an increased risk for incident hypertension (adjusted relative ratio, 1.41, 95 % CI 1.23–1.58).These effects were significantly larger in younger populations and tended to be larger in women, and the effect may be larger among African American individuals, concluding that hyperuricemia is associated with an increased risk for incident hypertension, independent of traditional hypertension risk factors [25]. Similarly, a recent meta-analysis consisting of 25 studies with a recruitment of 97,824 individuals showed a modest link between hyperuricemia and incident hypertension suggesting that hyperuricemia was associated with a higher risk of incident hypertension [30].

Our present study confirms previous studies that hyperuricemia, per se, worsened hypertension but also act as a trigger in its first onset. There were a small number of overlapping participants (13 individuals) who developed both CKD and hypertension at year 4, but we feel that this concomitance might not affect the positive link between the development hypertension and UA level.

A prospective cohort study recruiting 49,413 Japanese workers with a 7-year follow-up showed hyperuricemia has a strong association with the risks of death in all causes, CHD, stroke, liver disease and CKD suggesting that high UA appeared to be a considerable risk factor for reduced life expectancy [31].

## Conclusions

The present trial shows that high UA levels are associated with the new development of hypertension, but not with the incidence of CKD. Whether lowering of UA levels in hyperuricemic patients is beneficial or not remains to be investigated in the future.
